# Disruption of *yellow-e3* impairs both adult molting and cuticular melanization in the honeybee (*Apis mellifera*)

**DOI:** 10.1186/s12983-026-00606-5

**Published:** 2026-03-27

**Authors:** Yunxi Fu, Qiufang Li, Yu Lai, Shengli Wu, Linyan Tian, Shiwen Zhou, Liqiang Liang, Shangning Yang, Yulu Yi, Ping Zhao, Zhiguo Li, Songkun Su, Hongyi Nie

**Affiliations:** 1 College of Bee Science and Biomedicine, Fujian Agriculture and Forestry University, Fuzhou, 350002 China; 2 Enshi Tujia and Miao Autonomous Prefecture Academy of Agricultural Sciences, Enshi, 445000 China; 3 College of Animal Sciences, Fujian Agriculture and Forestry University, Fuzhou, 350002 China; 4 State Key Laboratory of Resource Insects, Biological Science Research Center, Southwest University, Chongqing, 400715 China; 5 Shandong Provincial Key Laboratory of Animal Cells and Developmental Biology, School of Life Sciences, Shandong University, Qingdao, 266237 China

**Keywords:** *Apis mellifera*, *Yellow-e3*, Molting, Eclosion rate, CRISPR/Cas9, RNAi

## Abstract

**Background:**

The insect *yellow* gene family plays crucial roles in cuticle pigmentation, waterproofing, courtship, molting, and eggshell development. However, the specific physiological function of *yellow-e3* remains unclear.

**Results:**

In this study, we first generated *yellow-e3* mutant drones using CRISPR/Cas9, achieving mutation rate of 87.4%, primarily (61.6%) comprising 5-bp deletions. But no discernible phenotypic differences were observed in these mutants. We therefore performed RNAi knockdown by injecting *yellow-e3* siRNA into 2-day-old pupae, which significantly reduced eclosion rate (36.14 vs. 72.53% in controls). Individuals that failed to eclose were arrested during molting, unable to shed the pupal cuticle, and exhibited overall yellow cuticles. Eclosed adults, by contrast, showed normal cuticle coloration. Further analysis revealed that non-eclosed individuals had significantly upregulated expression of ecdysone signaling genes *USP* and *E75*, but downregulated *E74*, accompanied by markedly reduced expression of melanin synthesis genes (*TH*, *yellow-y*, and *tan*). These changes ultimately disrupted melanin deposition, leading to cuticular yellowing.

**Conclusions:**

We propose that genetic compensation may conceal phenotypic defects in CRISPR-generated mutants. This study provides the first evidence that silencing *yellow-e3* in *A. mellifera* disrupts molting, reduces the eclosion rate, and inhibits cuticular melanization.

**Supplementary Information:**

The online version contains supplementary material available at 10.1186/s12983-026-00606-5.

## Background

The *yellow* gene family comprises ten subgroups, including yellow-y, yellow-b, yellow-c, yellow-d/e3, yellow-e, yellow-f, yellow-g/g2, yellow-h, yellow-x, and a hymenopteran-specific clade encoding Major Royal Jelly Proteins (MRJPs) [1]. This gene family has been extensively investigated across various insect orders, such as Diptera [2–4], Lepidoptera [5–7], Coleoptera [8, 9], Hymenoptera [10, 11], Hemiptera [12, 13], Orthoptera [14] and Blattodea [15]. Homologs of *yellow* genes have also been identified in bacteria, fungi and non-insect eukaryotes [16].

In insects, the *yellow* gene family is primarily known for its role in cuticular pigmentation. Among its members, *yellow-y* is the most extensively studied and has been shown to be essential for melanogenesis in Diptera, Lepidoptera, Hymenoptera, Coleoptera and Hemiptera [3, 11, 12, 17, 18]. Other members of the *yellow* family also contribute to pigmentation. In *Drosophila*, *yellow-f* regulates melanization in larval and pupal stages, while *yellow-f2* is involved in melanin biosynthesis in the late pupal and adult stages [19]. In butterflies, the *yellow-d* mutation enhances orange-brown pigmentation in specific regions of the ventral forewing [20]. The *yellow-e* gene is responsible for reddish-brown pigmentation in the larval head cuticle and anal plates of the *Bombyx mori* mutant *bts*/*bts2*, contrasting with the white coloration in wild-type individuals [21]. RNAi-mediated knockdown of *yellow-e* results in darker body pigmentation in adult *Tribolium castaneum* [8]. In *Henosepilachna vigintioctopunctata*, silencing of *yellow-b*, *yellow-e*, and *yellow-h* induced pigment accumulation, while suppression of *yellow-c* attenuated black coloration [9].

Beyond pigmentation, *yellow* gene family members are involved in diverse physiological processes in insects, including cuticle waterproofing, courtship behavior, oviposition, molting, and chorion development. For instance, loss of *yellow-e* function in* T. castaneum* leads to lethal entrapment within the pupal cuticle, a phenotype that can be rescued under high-humidity [8]. In *Aedes albopictus*, *yellow-g* and *yellow-g2* are required for egg desiccation resistance [22], and newly hatched G1 *yellow-y* mutant larvae of *Agrotis ipsilon* exhibit a dehydration phenotype [6]. Additionally, *yellow-y* modulates male courtship behavior in *Drosophila melanogaster* and German cockroach (*Blattella germanica*) [2, 15, 23]. In German cockroach females, *yellow-y* silencing reduces oviposition capacity [15]. Although *yellow-f* in *T. castaneum* does not influence pupal and adult pigmentation, it is essential for adult eclosion [18]. In *Drosophila*, *yellow-g* is expressed in follicle cells and is essential for eggshell formation [24], while in *T. castaneum*, *yellow-g* and *yellow-g2* are specifically expressed in the ovarioles of adult females, and are required for fecundity and egg chorion rigidity [1].

In *A. mellifera*, ten *yellow* genes (*yellow-y*, *yellow-b*, *yellow-e*, *yellow-e3*, *yellow-f*, *yellow-g*, *yellow-g2*, *yellow-h*, *yellow-* *x1* and *yellow-* *x2*) and ten MRJPs (*MRJP1-9* and *MRJP‐ψ*) have been identified [10, 25]. To date, only the *yellow-y* gene has been confirmed to participate in melanin synthesis [11], consistent with its orthologs in other insects; the functions of other *yellow* family members in *A. mellifera* remain largely unknown. Interestingly, *yellow-e3* and *yellow-h* directly flank the MRJP array, and *yellow-e3* exhibits a highly similar intron–exon structure to MRJP genes, suggesting that *yellow-e3* is the best candidate for the progenitor of all MRJP genes [10]. Although *yellow-e3* has been identified in *D. melanogaster* and *T. castaneum* [9], its precise physiological roles remain unclear. In this study, we employed CRISPR/Cas9 and RNAi to investigate the function of *yellow-e3* in *A. mellifera*. Our results demonstrate that this gene plays critical roles in adult molting and cuticular melanization, providing new insights into its functional significance in the honeybee (*A. mellifera*).

## Methods

### Sample collection

Colonies of *A. mellifera* (Fengqiang No.1 western honeybees) were raised at the apiary of the College of Bee Science and Biomedicine, Fujian Agriculture and Forestry University, Fuzhou, China. To obtain freshly laid eggs (within 2 h post-oviposition) for microinjection, the queen was confined to a comb containing empty cells for 2 h using an excluder cage (7.2 × 5.1 × 2.2 cm). The frame containing newly laid eggs was promptly transferred to a laboratory (30 °C), where eggs were grafted onto wax strips at a 15° angle using moving pipettes. For temporal expression analysis, samples from various developmental stages, including eggs (1- to 3-day-old), larvae (1-, 3- and 5-day-old), 1-day-old pre-pupae, pupae (0-, 2-, 4-, 6-, and 8-day old), and adults (newly emerged bees, 10-day-old nurses and 21-day-old foragers), were collected by controlling the queen’s egg-laying timing according to a previously established method [11]. To analyze expression profiles in newly emerged bees, diverse tissues, including deantennated head, antennae, thorax, abdomen (without gut and sting), gut, sting, wing and leg, were dissected on dry ice.

### Bioinformatics analysis

The yellow-e3 protein sequence was analyzed using the ExPASy ProtParam tool (http://web.expasy.org/protparam/) to predict its theoretical molecular mass and isoelectric point. The signal peptide, hydrophobicity, domain and transmembrane region were predicted using SignalP 4.1 (http://www.cbs.dtu.dk/services/SignalP/), ProtScale (http://web.expasy.org/protscale/), SMART (http://smart.embl-heidelberg.de/), and TMHMM 2.0 (http://www.cbs.dtu.dk/services/TMHMM/), respectively. Post-translational modification sites were predicted using: Phosphorylation sites (NetPhos 3.1; https://services.healthtech.dtu.dk/services/NetPhos-3.1/), O-glycosylation sites (NetOGlyc 4.0; https://services.healthtech.dtu.dk/services/NetOGlyc-4.0/), and N-linked glycosylation sites (NetNGlyc 1.0; http://www.cbs.dtu.dk/services/). Sequence identity was analyzed using Clustal Omega (https://www.ebi.ac.uk/Tools/msa/clustalo/). Yellow-e3 homologs in other insects were identified via NCBI BLASTp using the *A. mellifera* yellow-e3 sequence as queries. A phylogenetic analysis of *A. mellifera* yellow-e3 and homologs from *Apis cerana*, *Apis dorsata*, *Apis florea*, *Bombus terrestris*, *Megachile rotundata*, *Polistes dominula*, *Solenopsis invicta*, *Linepithema humile*, *Nasonia vitripennis*, *Acyrthosiphon pisum*, *Nilaparvata lugens*, *Culex quinquefasciatus*, *Plutella xylostella*, *Bombyx mori*, *Helicoverpa armigera*, *Drosophila melanogaster*, and *Tribolium castaneum* was conducted in MEGA 11.0. Phylogenies were constructed using the maximum likelihood method with a bootstrap test using 1000 replications.

### Real-Time Quantitative PCR (qPCR)

Specimens representing various developmental stages and tissues/parts from newly emerged workers were collected in Trizol. Total RNA was isolated using a total RNA kit according to the manufacturer’s instructions. RNA quality and concentration were assessed using agarose gel electrophoresis and spectrophotometry (Nanodrop 2000, Thermo Fisher Scientific). cDNA was synthesized from 1 μg of total RNA using HiScript II Q RT SuperMix for qPCR (Vazyme Biotech, Nanjing, China) following the manufacturer’s instructions. Gene-specific primers were designed by Primer Premier 5 and listed in Table S1. qPCR was performed on the CFX384 Touch Real-Time PCR Detection System (Bio-Rad, USA) using ChamQ SYBR Color qPCR Master Mix (Vazyme Biotech, Nanjing, China). The PCR procedures were 95 °C for 3 min, followed by 40 cycles at 95 °C for 10 s and 60 °C for 30 s. The relative gene expression level was calculated by the 2^−ΔΔCt^ method, with *A. mellifera actin* (NM_001185146.1) serving as the internal reference gene. Each group has three biological replicates and was repeated in triplicate. Data are presented as the mean ± SEM, and statistical significance was evaluated by one-way ANOVA or Student’s t-test using GraphPad Prism 8.0.

### sgRNA synthesis and its verification in vitro

The genomic sequence of *yellow-e3* was delivered to CRISPR Optimal Target Finder (http://targetfinder.flycrispr.neuro.brown.edu/), and its sgRNA were designed. sgRNA was synthesized via PCR as our previously described method [11]. The efficiency of sgRNA cleavage was assessed in vitro. Given the proximity of the three sgRNA target sites within the genomic region, we amplified a single PCR fragment encompassing all three sites using flanking primers. The fragments were incubated in a cleavage reaction mixture containing: 1 μL of PCR fragments, 1 μL sgRNA (100 ng/μL), 1 μL Cas9 protein (100 ng/μL, Takara), 2 μL 10 × Cas9 buffer, and 15 μL ddH_2_O. The reaction conditions were as follows: 37 °C for 30 min and 70 °C for 10 min. A negative control reaction replaced the sgRNA with an equivalent volume of ddH_2_O. And its cleavage efficiency was monitored via 1% agarose gel electrophoresis.

### Injection and rearing

Newly laid eggs (within 2 h after oviposition) were fixed on a wax strip at a 15° angle and injected with a mixture of sgRNA (375 ng/μL) and Cas9 protein (300 ng/μL) using a microinjection device (PLI-100, Medical Systems Corporation) according to our previously described method [11]. Immediately after injection, the eggs were transferred to an incubator maintained at 34 °C and 85% RH. Upon hatching, queen-destined larvae were grafted gently into queen cell cups and placed into a queenless colony, where they received abundant fresh royal jelly from nurse bees [26]. Emerged queens were subsequently introduced into queenless nucleus hives. To generate edited drones, queens were treated with CO_2_ to induce the production of unfertilized eggs [26], which developed into drones. Colonies were set with a queen excluder at the entrances to avoid edited individuals escaping into nature.

### RNAi treatment

Specific siRNA targeting *yellow-e3* were designed using siDirect 2.0 (http://siDirect2.RNAi.jp/) based on sequence specificity principles (Table S1) and commercially synthesized. For RNAi-mediated knockdown, different doses of *yellow-e3* siRNA (500 ng and 1000 ng) were microinjected into the dorsal thorax of 2-day-old pupae. After injection, the individuals were maintained in an incubator at 34 °C and 75% RH. RNAi bioassays were performed with five biological replicates. Injected pupae were monitored daily until adult eclosion to assess phenotypic effects. The mRNA levels of *yellow-e3* were measured using qPCR in both the experimental and control groups. Additionally, in individuals derived from 2-day-old pupae injected with *yellow-e3* siRNA, we examined the expression of other members of the *yellow* gene family and key nuclear receptor genes in the ecdysone signaling pathway, comparing those that failed to eclose completely (siRNA-*yellow-e3*(−)) with those that eclosed successfully (siRNA-*yellow-e3*( +)). To further investigate the role of *yellow-e3* in melanin synthesis in *A. mellifera*, we analyzed the expression levels of key melanin synthesis pathway genes in siRNA-*yellow-e3*(−) versus siRNA-*yellow-e3*( +) individuals.

## Results

### Bioinformatic characterization of *A. mellifera* yellow-e3

**Fig. 1:**
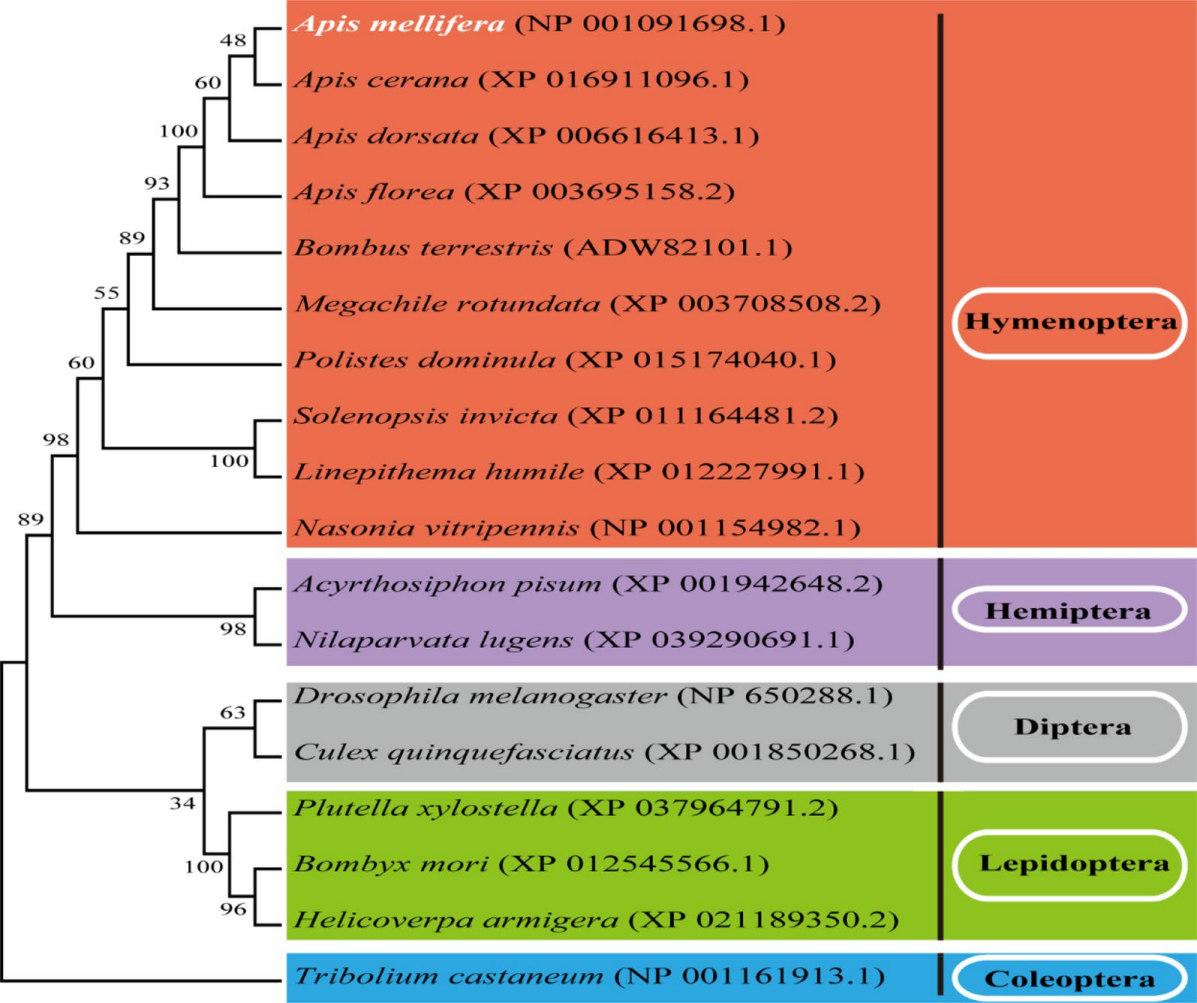
Phylogenetic tree of insect yellow-e3

Using cDNA from newly emerged workers as a template, PCR amplification yielded a *yellow-e3* product of approximately 1700 bp, confirmed by sequencing. Bioinformatics analysis revealed a full-length CDS sequence of 1281 bp, consistent with the predicted *yellow-e3* sequence (NCBI accession: LOC413894). This CDS encodes a 426 amino acid protein harboring an MRJP domain (residues 123–414). The predicted protein has a theoretical molecular mass of 48.5 kDa and an isoelectric point (pI) of 5.58. Amino acid composition analysis indicates that yellow-e3 is a hydrophilic protein containing a signal peptide but lacking transmembrane region. The yellow-e3 protein contains 1 O-glycosylation site (residue 293), 3 N-glycosylation sites (residues 297, 285 and 394), and 43 phosphorylation sites. The yellow-e3 protein of *A. mellifera* shares 29.95–95.28% amino acid identity with its homologs in other insects (Table S2). Phylogenetic analysis showed that *A. mellifera* yellow-e3 was well clustered with other hymenopteran insect homologs, particularly *Apis cerana* (Fig. 1), suggesting that they may have similar functions.

### Expression patterns of *A. mellifera yellow-e3* gene

**Fig. 2:**
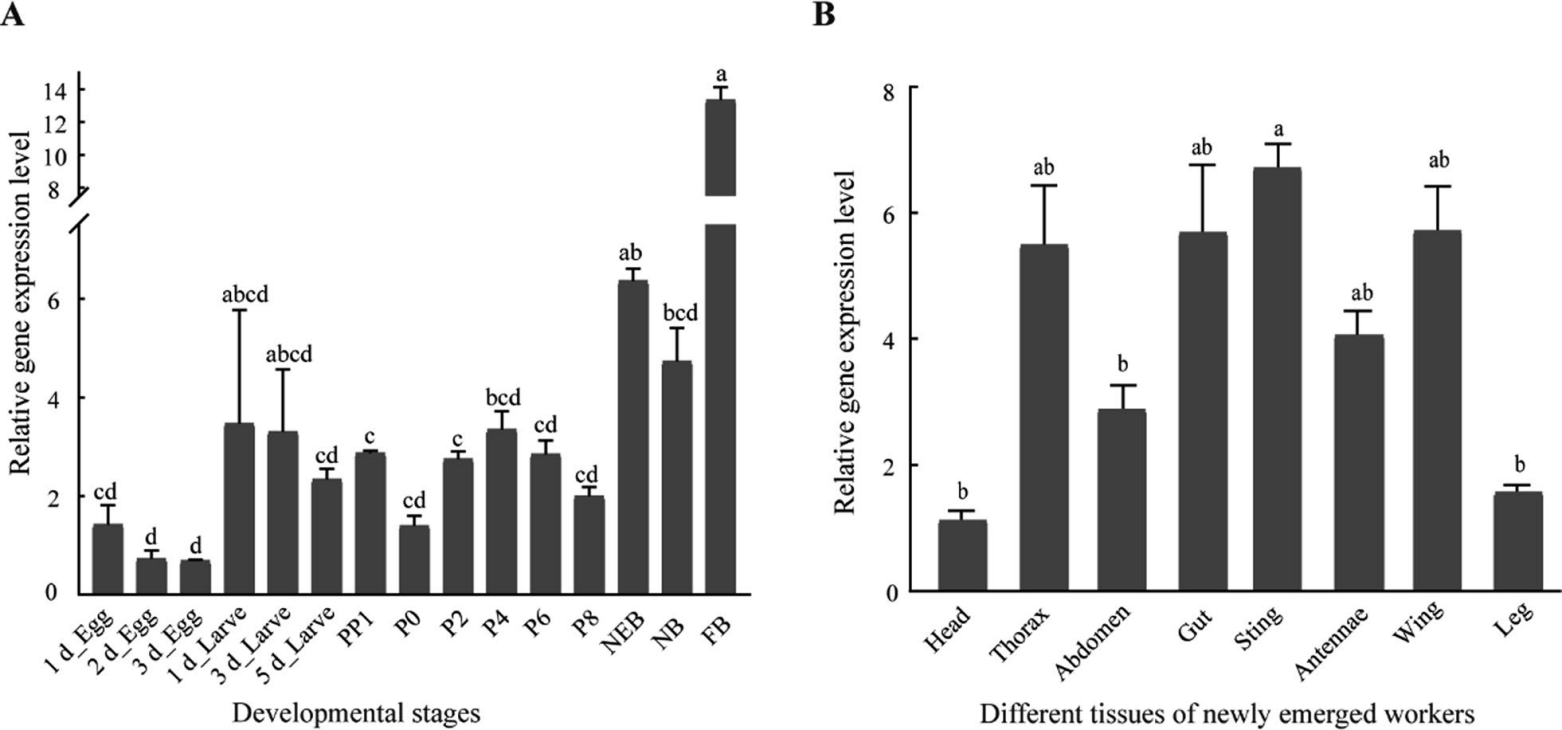
Spatio-temporal expression of *yellow-e3* in *A. mellifera*. **A** Temporal expression profile across development stages. Developmental stages: 1 d_Egg, 2 d_Egg and 3 d_Egg (1-, 2-, and 3-day-old egg); 1 d_Larvae, 3 d_Larvae and 5 d_Larvae (1-, 3-, and 5-day-old larvae); PP1 (day 1 pharate pupae); P0, P2, P4, P6 and P8 (0-, 2-, 4-, 6- and 8-day-old pupae); NEB (newly emerged bees), NB (nurses) and FB (foragers). **B** Tissue-specific expression in newly emerged workers. Analyzed tissues: head (antennae removed), thorax, abdomen (no sting, venom gland and gut), antennae, gut, sting (with venom gland), wing and leg. Different lowercase letters indicate significant differences, as determined by one-way ANOVA followed by Tukey’s test (*P* < 0.05). For both panels, different lowercase letters indicate a statistically significant difference (*P* < 0.05) in relative gene expression levels between the corresponding groups, while the same lowercase letter or overlapping letter combinations mean no significant statistical difference (*P* > 0.05) between the groups

The stage expression profiles of *yellow-e3* showed that it was expressed throughout the whole development stage (Fig. 2A). Its expression was weak at the egg stages with falling tendency, and increased at the larvae and pre-pupal stages. Notably, expression peaked in 4-day-old pupae, with significant fluctuations observed throughout the pupal stage. Following adult emergence, *yellow-e3* transcript levels rose sharply in newly emerged workers and reached their highest abundance in foragers. Tissue expression patterns of *A. mellifera yellow-e3* in eight tissues/part (Head, Thorax, Abdomen, Gut, Sting, Antennae, Wing, Leg) collected from newly emerged workers was determined (Fig. 2B). The level of *yellow-e3* expression was high in the sting, wing, gut and thorax, but lower in the antennae, abdomen, leg and head.

### CRISPR/Cas9 induced mutagenesis at* A. mellifera yellow-e3* loci

**Fig. 3:**
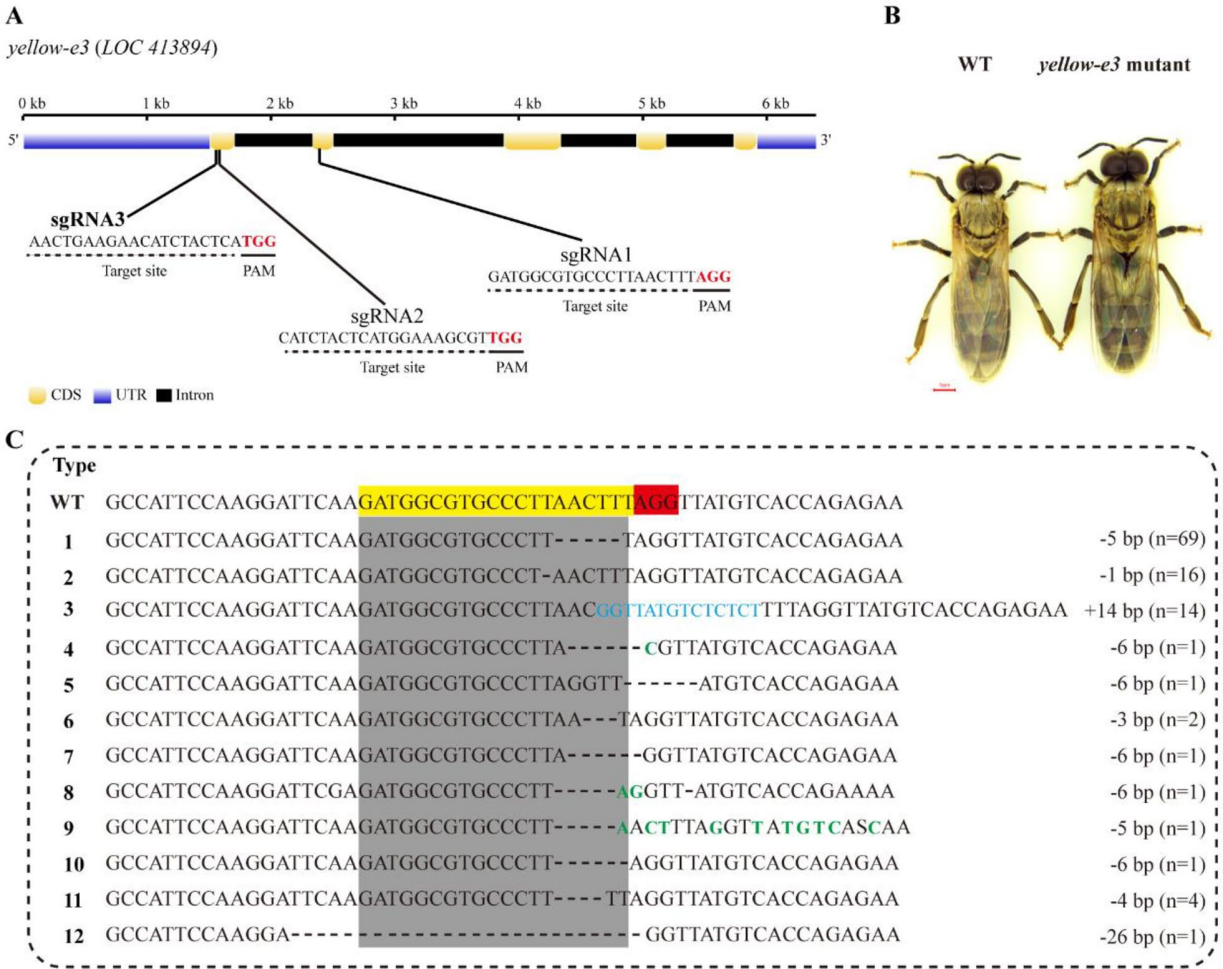
Targeted mutation of *yellow-e3* drones using CRISPR/Cas9. **A** Schematic of *A. mellifera yelllow-e3* showing sgRNA target sites. Blue, yellow and black boxes represent untranslated region, exons and introns, respectively. The protospacer adjacent motif (PAM) sequences are highlighted in red. **B** Photograph comparison of representative wild-type (WT) and *yelllow-e3* mutant drones from edited queens. **C** Sequences surrounding the sgRNA site were validated by PCR amplification and sequencing. The target site is highlighted in yellow; the PAM is highlighted in red. Dashed lines represent the deleted bases; the blue and green nucleotides represent substitutions and insertions, respectively. The net change in length is marked on the right of each sequence. “n” represents the number of mutation types

**Fig. 4:**
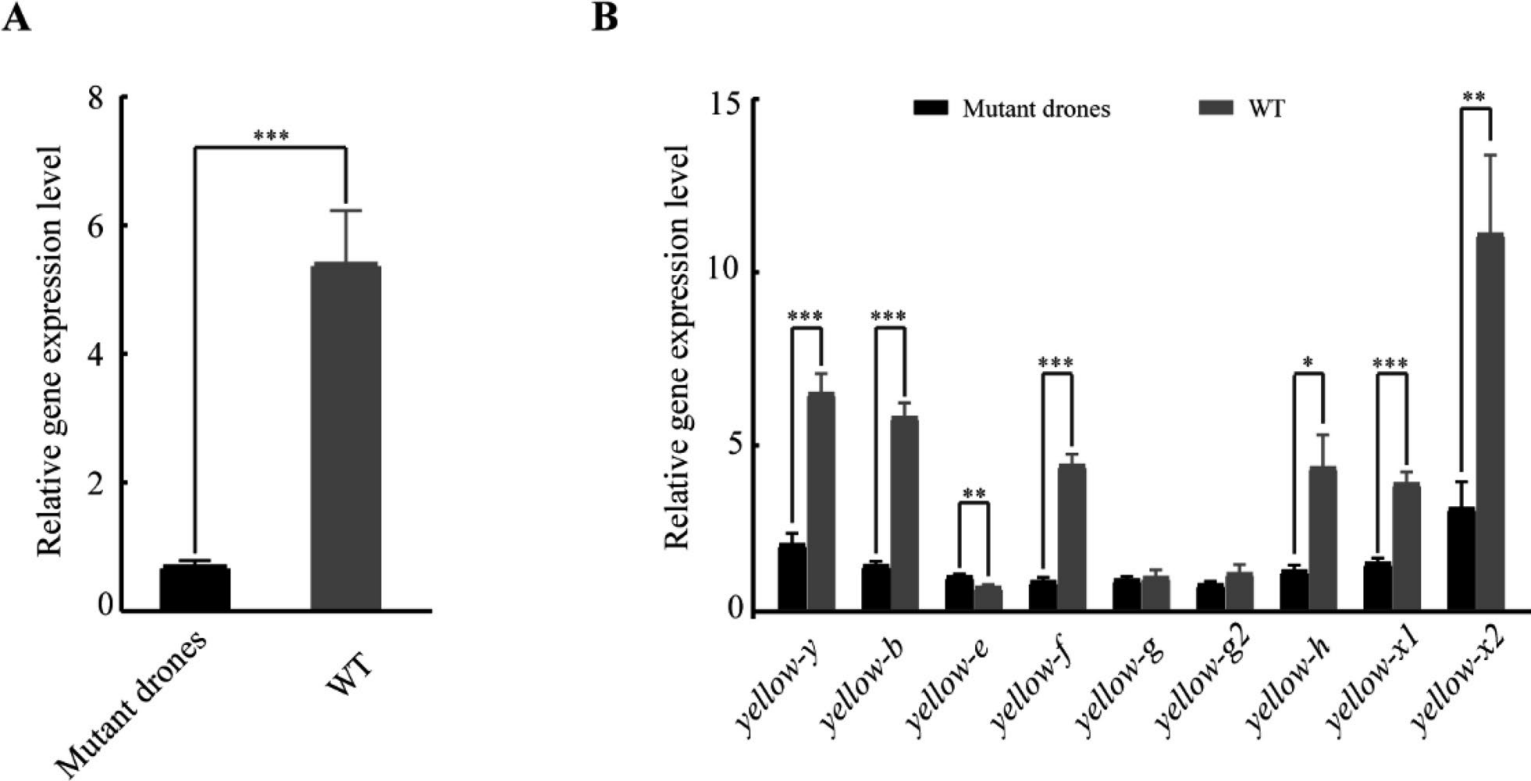
Expression analysis of *yellow* family genes in *yellow-e3* mutant drones. **A** Expression profiles of *yellow-e3* in mutant drones. **B** Relative expression levels of other yellow family homologous genes in *yellow-e3* mutant drones. Data represent mean ± SEM from three biological replicates. Independent sample *t*-test was used to assess statistical differences in gene expression levels, where * means *P* < 0.05, ** indicates *P* < 0.01, and *** represents *P* < 0.001

Three sgRNAs (sgRNA1, sgRNA2 and sgRNA3) were designed to target exons 1 and 2 of the *yellow-e3* gene (Fig. 3A). To test the activity of these sgRNAs complexed with Cas9 protein, PCR products harboring three targets were digested in vitro under a mixture of each sgRNA and Cas9 protein. Agarose gel electrophoresis showed that the original PCR products were almost digested, and produced expected bands in the verified system with sgRNA1 and Cas9 protein, suggesting that sgRNA 1 possesses higher efficiency than the others (Fig. S1). Consequently, sgRNA 1 was selected for subsequent embryo microinjection. As a control, a mixture of EGFP sgRNA and Cas9 protein was injected into 30 freshly laid eggs. Of these, 28 eggs hatched after three days, 24 larvae developed into pre-pupa, 14 pre-pupae developed into pupa, and finally 6 virgin queens emerged successfully. In parallel, we injected 100 eggs with a mixture of Cas9 protein and *yellow-e3* sgRNA 1, yielding 39 hatched larvae. These larvae were transferred into queen cell cups and returned to the original colony. After queen rearing, eleven virgin queens emerged. Seven of these queens laid unfertilized eggs, which successfully developed into adult drones. However, no obvious phenotypic differences were observed compared to the control group upon emergence (Fig. 3B). To determine whether the *yellow-e3* sgRNA 1 target site was edited in newly emerged offspring drones, we genotyped the target region using PCR and sequencing. Analysis confirmed that these queens produce genetically edited offspring drones with an average editing efficiency of 87.4% (Table S3). The edits included deletion, insertion and substitution surrounding the target sites (Fig. 3C). Analysis of 112 edited progenies revealed that 69 (61.6%) carried the predominant 5-bp deletion (Fig. 3C). This mutation is expected to cause a frameshift, which is predicted to introduce a premature stop codon at amino acid position 112. qPCR analysis showed that expression of *yellow-e3* was significantly reduced in mutant drones harboring the 5-bp deletion compared to the controls (Fig. 4A). Further expression profiling of other *yellow* family genes in these mutants indicated that *yellow-e* was significantly upregulated, while *yellow-y*, *yellow-b*, *yellow-f*, *yellow-h*, *yellow-* *x1*, and *yellow-* *x2* were significantly downregulated (Fig. 4B).

### *yellow-e3* significantly decreased the eclosion rate of *A. mellifera*

**Fig. 5:**
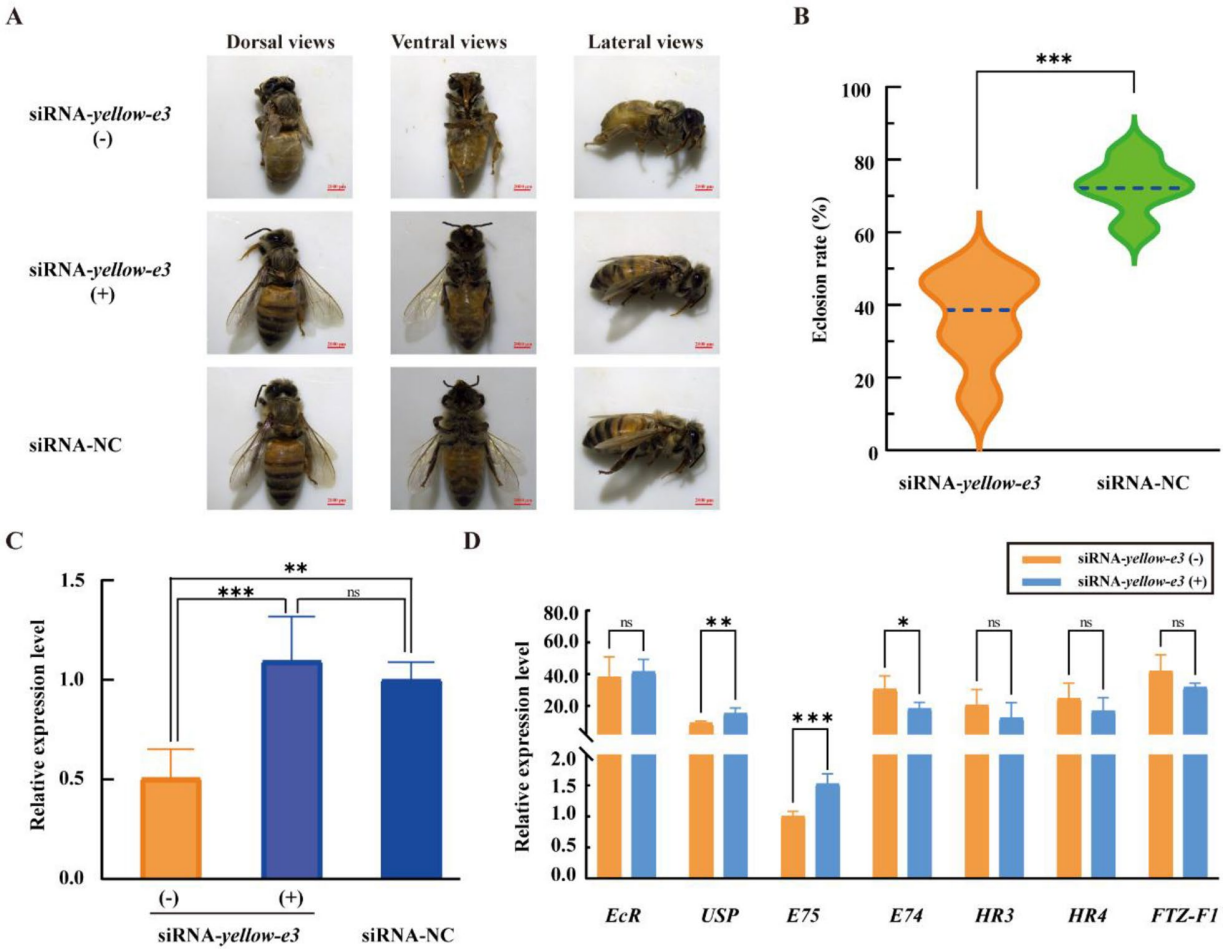
RNAi-mediated knockdown of *yellow-e3* gene in honeybee pupae. **A** Phenotypes of non-eclosed pharate adults (siRNA-*yellow-e3* (−)), successfully eclosed adult (siRNA-*yellow-e3* ( +)) and control (siRNA-NC). **B** Eclosion rates following microinjection of *yellow-e3* siRNA into 2-day-old pupae. NC represents negative control. **C** Relative expression level of *yellow-e3* after silencing. (−) and ( +) represent individuals that failed to eclose completely or achieved successful eclosion when 2-day-old pupae were injected with *yellow-e3* siRNA, respectively. The bar represents mean ± SE (n = 5/6); bars labeled with asterisks are significantly different (one-way ANOVA; **P < 0.01; *** *P* < 0.001; ns: no significant). **D** Effect of *yellow-e3* silencing on ecdysone signaling pathway gene expressions. The gene expressions in the ecdysone signaling pathway were detected by qPCR in individuals that failed to eclose completely or achieved successful eclosion. Bars represent mean ± SEM (n = 5); bars labelled with asterisks are significantly different (t-test; *P < 0.05; ** *P* < 0.01; ***P < 0.001; ns: no significant)

First, to exclude the potential influence of RNAi off-target effects, we injected three non-overlapping siRNAs (siRNA1, siRNA2, and siRNA3) designed against the *yellow-e3* gene into 2-day-old pupae, observed their phenotypes upon development to the eclosion stage, and subsequently calculated the eclosion rates. The results showed that all three siRNAs disrupted the normal molting process of the pupae, leading to a significant decrease in the eclosion rate (Fig. S2). Moreover, as the concentration of siRNA increased, the eclosion rate exhibited a gradual decline (Fig. S2). To further validate the molecular changes resulting from *yellow-e3* gene silencing and to ensure the reliability of the results, we subsequently conducted five independent biological replicates using *yellow-e3* siRNA1. The experimental group (siRNA-*yellow-e3*; 231 two-day-old pupae) was injected with *yellow-e3* siRNA1, while the control group (siRNA-NC; 157 two-day-old pupae) received negative control siRNA. Throughout the pupal stages, daily observations revealed no significant morphological or developmental differences between the groups (Fig. S3). Upon reaching the eclosion stage, we assessed the ability of individuals in both groups to molt and eclose normally. Most individuals in the experimental group failed to fully eclose from the pupal exuviae, remaining trapped as pharate adults (Fig. 5A). In the experimental group, 84 individuals successfully eclosed, with an eclosion rate of 36.14% (Fig. 5B and Table S4). In contrast, 113 individuals eclosed in the control group, achieving an eclosion rate of 72.53% (Fig. 5B and Table S4). The significantly lower eclosion rate in the experimental group indicates that silencing *yellow-e3* expression disrupts the adult molting process, thereby reducing eclosion success. We also observed that non-eclosed individuals in the siRNA-*yellow-e3* group exhibited an overall yellowish coloration, whereas eclosed individuals resembled those in the siRNA-NC group, displaying predominantly black pigmentation (Fig. 5A). qPCR analysis was performed to determine whether this color difference correlated with *yellow-e3* gene expression levels. The results revealed significantly lower *yellow-e3* gene expression in the non-eclosed siRNA-*yellow-e3* group (siRNA-*yellow-e3*(−)) compared to both the eclosed siRNA-*yellow-e3* group (siRNA-*yellow-e3*( +)) and the siRNA-NC group (Figs. 5C and S4A). Furthermore, we observed that the expression levels of other members of the *yellow* gene family, including *yellow-y*, *yellow-b*, *yellow-f*, and *yellow-* *x1*, were significantly reduced in individuals (siRNA-*yellow-e3*(−)) that failed to eclose (Fig. S4B). This expression pattern is consistent with the trends observed in *yellow-e3* knockout drone mutants (Fig. 4B).

Given that *EcR*, *USP*, *E75*, *E74*, *HR3*, *HR4*, and *FTZ-F1* are key nuclear receptor genes in the ecdysone signaling pathway in *Bombyx mori* [27], we examined the expression levels of their homologous genes in siRNA-*yellow-e3*(−) versus siRNA-*yellow-e3*( +) individuals. It showed that the expression of *USP* and *E75* was significantly reduced in the siRNA-*yellow-e3*(−) group, while that of *E74* was significantly elevated (Fig. 5D). This suggests that silencing the *yellow-e3* gene in *A. mellifera* may impair molting by regulating the expression of *USP*, *E75* and *E74* within the ecdysone signaling pathway, resulting in abnormal eclosion.

### *yellow-e3* mediates melanin biosynthesis in adult *A. mellifera*

**Fig. 6:**
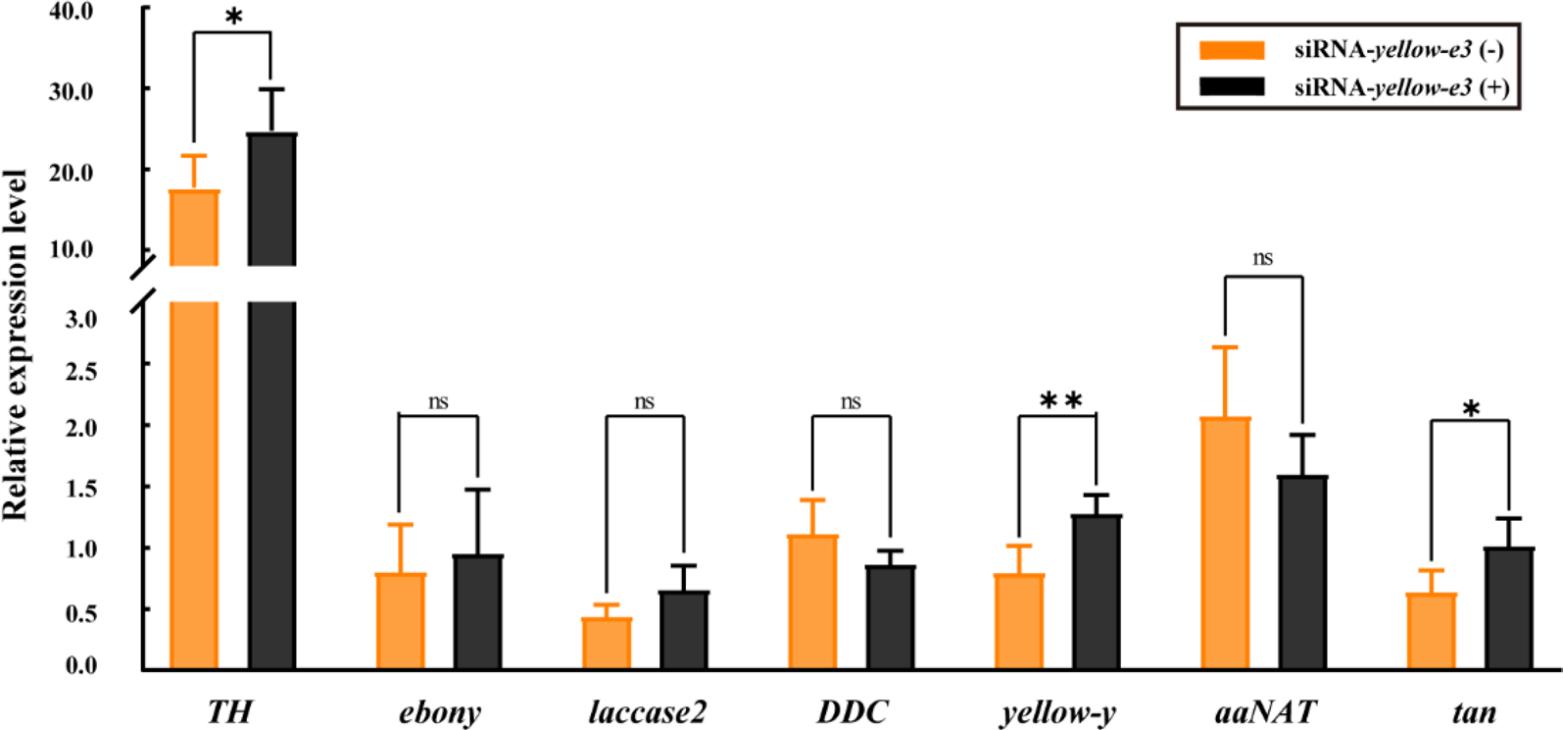
Relative expression levels of melanin synthesis pathway genes in individuals that failed to completely eclose or successfully eclosed following injection of *yellow-e3* siRNA into 2-day-old pupae. Bar represent mean ± SEM (n = 5). Significant differences between groups are indicated by asterisks (t-test; *P < 0.05; **P < 0.01; ns: no significant)

The siRNA-*yellow-e3* group showed distinct phenotypic differences between non-eclosed(siRNA-*yellow-e3*(−)) and eclosed adults (siRNA-*yellow-e3*( +)) (Fig. 5A). We further examined the expression differences of seven key melanin pathway genes (*TH*, *ebony*, *laccase 2*, *DDC*, *yellow-y*, *aaNAT*, and *tan*) between these two groups. The expression of three genes (*TH*, *yellow-y*, and *tan*) were significantly decreased in non-eclosed adults, whereas the remaining genes showed no significant differences (Fig. 6).

## Discussion

In insects, the *yellow* gene family plays crucial roles not only in cuticle pigmentation but also in multiple additional physiological processes, such as waterproofing, courtship behavior, oviposition, molting, and chorion development [1, 8, 15, 17, 18]. However, the physiological function of *yellow-e3* remains uncharacterized in insects. In this study, we elucidated its function in *A. mellifera* using CRISPR/Cas9 and RNAi. Silencing the expression of *yellow-e3* significantly impaired adult eclosion rates and reduced cuticular melanin synthesis. These findings not only directly expand the known physiological roles of the *yellow* gene family in *A. mellifera*, but also provide valuable insights and a theoretical framework for elucidating the functions of *yellow-e3* in other insects.

*MRJP* genes, specific to the order Hymenoptera, exhibit diverse functions, including nutritional function, antibacterial activity, and learning ability [28]. In *A. mellifera*, ten *MRJP* genes are tandemly arrayed on chromosome 11, flanked by *yellow-e3* and *yellow-h* [28]. Phylogenetic analyses reveal that the *yellow* gene family forms a monophyletic clade distinct from *MRJPs*, with *yellow-e3* representing the most recent common ancestor of the *MRJP* cluster [10]. This suggests that *MRJPs* have likely evolved recently via rapid and repeated duplications of *yellow-e3*. However, the physiological function of the *yellow-e3* gene still remains unclear. Given that CRISPR/Cas9 gene editing provides a powerful genetic tool for validating gene function in *A. mellifera* [29, 30], we first employed this technology to generate *yellow-e3* mutant drones. The editing efficiency reached 87.4%, with 61.6% of mutant drones carrying a predominant 5-bp deletion at the target site (Fig. 3C and Table S2). This deletion induces a frameshift mutation predicted to create a premature stop codon at amino acid position 112. Considering the crucial role of the *yellow* gene family in cuticle melanization, we specifically examined whether mutants exhibited visible pigmentation defects. However, *yellow-e3* mutant drones showed no significant difference in body color compared to controls after eclosion. Targeted gene knockout failing to yield discernible phenotypes has been widely reported in zebrafish. For instance, zebrafish *egfl7* mutants generated using TALENs exhibited no apparent abnormalities, whereas knockdown caused severe vascular defects [31]. CRISPR/Cas9-generated *epoa* homozygous mutants developed normally, but morpholino-mediated knockdown resulted in severe anemia and pronephros defects [32]. TALEN-derived *capn3a* mutant with 14 bp deletion displayed a normal liver phenotype, in contrast to knockdown individuals showing reduced liver size [33]. *Nid1a* knockout mutants exhibited a transient short-body phenotype at 1–3 days post-fertilization (dpf), which was progressively rescued by 4–5 dpf, while *nid1a* knockdown embryos consistently exhibited shortened body length throughout development [34]. The disparity in phenotypes caused by gene knockout versus knockdown might stem from knockout-induced genetic compensation response, which masks observable phenotypic effects. Therefore, we also employed RNAi-mediated silencing to suppress the *yellow-e3* gene in *A. mellifera*. This resulted in a mean eclosion rate of 36.14%, significantly lower than the control group (Fig. 5B and Table S3). Furthermore, we observed that non-eclosed adults in the experimental group exhibited an overall yellower cuticle coloration. In contrast, successfully eclosed individuals within the experimental group displayed a darker pigmentation similar to the control group (Fig. 5A). Studies have demonstrated that triggering the genetic compensation response (GCR) through gene knockout requires the simultaneous fulfillment of two conditions: (i) generation of a premature termination codon (PTC) in the target gene and (ii) upregulated expression of homologous genes [33]. In our study, we detected a high proportion of mutations in *yellow-e3* knockout mutants, with 61.6% being 5-bp deletions (Table S2 and Fig. 3C). These deletions are predicted to introduce a PTC at amino acid position 112. Concurrently, we observed significant upregulation of *yellow-e* genes in the mutants, within the *yellow* gene family comprising 10 members (Fig. 4B). We therefore hypothesize that the *yellow-e3* knockout triggered a GCR, resulting in the absence of overt phenotypic changes. Since our analysis was limited to newly emerged mutants, it is possible that compensatory effects are more pronounced during the pupal stage. To address this, we will examine the expression of the *yellow* gene family across various pupal developmental stages, aiming to comprehensively identify potential genetic compensators for *yellow-e3*. Furthermore, we will employ gene knockout approaches to generate double knockouts, thereby elucidating the molecular mechanisms underlying compensation.

In arthropods, molting constitutes a critical physiological process that facilitates growth and development through new cuticle synthesis and old cuticle shedding [35, 36]. Thus, successful molting is essential for growth, development, reproduction, and survival [37]. Previous studies indicate that the *yellow* gene family plays crucial role in insect molting. For example, *yellow-y* knockout in *Spodoptera litura* prevents normal molting in 6-day-old larvae [38]; *yellow-f* knockdown via RNAi in *T. castaneum* causes failed pupal cuticle shedding and entrapment death [18]. Similarly, our findings demonstrate that *yellow-e3* silencing in *A. mellifera* disrupted ecdysis completion, reducing the eclosion rate to 36.14% and ultimately arrested development at the pharate adult stage (Fig. 5A and B). Concurrently, we found that the expression of key nuclear receptor genes *USP* and *E75* in the ecdysone signaling pathway was significantly reduced in unemerged adults from the experimental group, whereas *E74* expression was significantly elevated. *EcR* silencing in *Drosophila* disrupts larval molting/metamorphosis and prevents pupation [39]. Similarly, *EcR* knockdown in *Helicoverpa armigera* causes molting defects and larval mortality [40]. *E75* suppression in *Sitobion avenae* nymphs increases mortality and molting deformities [41]. *E74B* silencing in *H. armigera* impaired larval molting and growth, resulting in over 60% larval mortality, and also severely disrupted the metamorphosis process, reducing both pupation and emergence rates [42]. We therefore hypothesize that *yellow-e3* silencing in *A. mellifera* disrupts the regulatory dynamics of *USP*, *E75*, and *E74* within the ecdysone signaling pathway, consequently impeding the molting process and ultimately suppressing adult emergence rates. These findings elucidate the physiological function of the *yellow-e3* gene in regulating the molting and eclosion in *A. mellifera*. Given that *A. mellifera* is a beneficial pollinator of critical agricultural importance, we recognize that any potential pest control applications must be considered with extreme caution regarding target specificity and ecological safety. Nevertheless, the high sequence conservation of yellow-e3 between *A. mellifera* and various pest insects (Fig. 1 and Table S2) raises the possibility that this gene may play similar functional roles in molting regulation in pest species. However, sequence conservation alone does not guarantee functional conservation, or address potential off-target risks to beneficial insects. Therefore, before any biocontrol applications can be considered, future studies would need to (1) establish direct functional evidence for *yellow-e3* in target pest species using species-specific RNAi approaches; (2) comprehensively evaluate sequence divergence between pests and beneficial species to identify target regions that could enable species-specific silencing; and (3) assess potential off-target effects on non-pest insects, particularly pollinators.

Melanin is the primary contributor to body coloration in adult *A. mellifera*, and the *yellow* gene family plays a critical role in melanin biosynthesis [11]. This study found that silencing *yellow-e3* resulted in a yellowish coloration of the cuticle in unemerged adults (Fig. 5A). Further analysis of the expression levels of key genes in the melanin pathway revealed that the expression of *TH*, *yellow-y*, and *tan* was significantly lower in unemerged adults compared to emerged adults (Fig. 6). Since *TH* is a key rate-limiting enzyme in the melanin pathway, its reduced expression led to decreased levels of its products, DOPA and dopamine [43]. Concurrently, *yellow-y* plays a crucial role in the synthesis of dopamelanin and dopamine melanin, and its knockout results in an overall yellowish body color in* A. mellifera* [11].Therefore, we hypothesize that in *yellow-e3*-silenced unemerged adults, reduced *TH* expression diminishes the synthesis of DOPA and dopamine, while decreased *yellow-y* expression impairs the synthesis of dopamelanin and dopamine melanin. This likely leads to a relative increase in the conversion of substrates to NADA, ultimately causing the entire cuticle to exhibit a yellowish coloration.

## Conclusion

In summary, we knocked out the *yellow-e3* gene in *A. mellifera* using CRISPR/Cas9 and obtained mutant drones. These mutants exhibited no discernible phenotypic abnormalities. In contrast, RNAi-mediated suppression of *yellow-e3* expression disrupted the pupal-adult transition, significantly reduced eclosion rates and reduced cuticular melanization in developmentally arrested pharate adults. We therefore propose that *yellow-e3* knockout triggers a genetic compensation response (GCR), thereby masking phenotypic consequences in mutants. Future studies should identify the specific compensatory homolog(s) for *yellow-e3*. Dual knockout of *yellow-e3* and its compensatory gene(s) will elucidate the molecular mechanism underlying GCR in this system.

## Supplementary Information

Additional file 1 (RAR 1549 KB)

Additional file 2 (DOCX 16 kb)

## Data Availability

All data supporting the conclusions of this article are included within the article and its additional files.
