# Supplementary material for: Disruption of yellow-e3 impairs both adult molting and cuticular melanization in the honeybee (Apis mellifera)
Source: Front Zool. 2026 Mar 27;23:18. doi: 10.1186/s12983-026-00606-5 (PMC13147567; doi:10.1186/s12983-026-00606-5)
Supplement: Supplementary file 2 — Additional file 2 (DOCX 16 kb) [file 12983_2026_606_MOESM2_ESM.docx]

**Supplementary material**

**Figure S1** **The cleavage efficiencies of three sgRNAs complexed with Cas9 protein were evaluated in an in vitro system.** NC denotes negative control group, where sgRNA was omitted and replaced with ddH_2_O. Underlined Number indicate the size of corresponding bands.

**Figure S2 Effects of three non-overlapping siRNAs targeting the *yellow-e3* gene in *Apis mellifera* on eclosion rate.** (A) Eclosion rate of 2-day-old *A. mellifera* pupae injected with different concentrations of negative control siRNA (siRNA-NC). (B–D) Eclosion rate of 2-day-old *A. mellifera* pupae injected with different concentrations of siRNA1-*yellow-e3*, siRNA2-*yellow-e3*, and siRNA3-*yellow-e3*, respectively.

**Figure S3** Morphological observation throughout the pupal stage when 2-day-old pupae were injected siRNA of *yellow-e3*.

**Figure S4** **Expression analysis of *yellow* family genes in *yellow-e3* siRNA treated individuals. (A) Expression profiles of *yellow-e3* in non-eclosed pharate adults (siRNA-*yellow-e3* (-)) and successfully eclosed adult (siRNA-*yellow*-e3 (+)). (B) Relative expression levels of other yellow family homologous genes in non-eclosed pharate adults (siRNA-*yellow-e3* (-)) and successfully eclosed adult (siRNA-*yellow*-e3 (+)).** Data represent mean±SEM from three biological replicates. Independent sample T-test was used to assess statistical differences in gene expression levels, where * means *P*<0.05, ** indicates *P*<0.01, and *** represents *P*<0.001; ns: no significant.

**Table S1** Primers used in this study.

**Table S2** Percent identity between the yellow-e3 of *Apis mellifera* and homologous genes in other insects.

**Table S3** Numbers and ratios of genome-edited offspring produced from seven queens that laid eggs.

**Table S4** Eclosion rates of workers following *yellow-e3* siRNA injection in 2-day-old pupae.
